# Impact of Soil Inoculation with *Bacillus amyloliquefaciens* FZB42 on the Phytoaccumulation of Germanium, Rare Earth Elements, and Potentially Toxic Elements

**DOI:** 10.3390/plants11030341

**Published:** 2022-01-27

**Authors:** Precious Uchenna Okoroafor, Lotte Mann, Kerian Amin Ngu, Nazia Zaffar, Nthati Lillian Monei, Christin Boldt, Thomas Reitz, Hermann Heilmeier, Oliver Wiche

**Affiliations:** 1 Institute of Biosciences, Interdisciplinary Environmental Research Centre, Technische Universität Bergakademie Freiberg, Leipziger Str. 29, 09599 Freiberg, Germany; lotte.mann@gmail.com (L.M.); kerian.amin-ngu@student-tu-freiberg.de (K.A.N.); nazia.zaffar@student.tu-freiberg.de (N.Z.); nthati-lillian.monei1@extern.tu-freiberg.de (N.L.M.); christin.boldt@ioez.tu-freiberg.de (C.B.); hermann.heilmeier@ioez.tu-freiberg.de (H.H.); oliver.wiche@ioez.tu-freiberg.de (O.W.); 2 Mining Department, Geology Institute, Tallinn University of Technology, 19086 Tallin, Estonia; 3 Department of Soil Ecology, Helmholtz Centre for Environmental Research–UFZ, Theodor–Lieser Str. 4, 06120 Halle, Germany; thomas.reitz@ufz.de

**Keywords:** *Bacillus amyloliquefaciens*, phytoextraction, potentially toxic elements, germanium, rare earth elements, bioinoculants

## Abstract

Bioaugmentation promises benefits for agricultural production as well as for remediation and phytomining approaches. Thus, this study investigated the effect of soil inoculation with the commercially available product RhizoVital^®^42, which contains *Bacillus amyloliquefaciens* FZB42, on nutrient uptake and plant biomass production as well as on the phytoaccumulation of potentially toxic elements, germanium, and rare earth elements (REEs). *Zea mays* and *Fagopyrum esculentum* were selected as model plants, and after harvest, the element uptake was compared between plants grown on inoculated versus reference soil. The results indicate an enrichment of *B. amyloliquefaciens* in inoculated soils as well as no significant impact on the inherent bacterial community composition. For *F. esculentum*, inoculation increased the accumulation of most nutrients and As, Cu, Pb, Co, and REEs (significant for Ca, Cu, and Co with 40%, 2042%, and 383%, respectively), while it slightly decreased the uptake of Ge, Cr, and Fe. For *Z. mays*, soil inoculation decreased the accumulation of Cr, Pb, Co, Ge, and REEs (significant for Co with 57%) but showed an insignificant increased uptake of Cu, As, and nutrient elements. Summarily, the results suggest that bioaugmentation with *B. amyloliquefaciens* is safe and has the potential to enhance/reduce the phytoaccumulation of some elements and the effects of inoculation are plant specific.

## 1. Introduction

Soil pollution majorly arises from the dumping of waste from natural or anthropogenic sources in soil, thereby causing undesirable impacts on the chemical, biological, and physical properties of air, soil, and water [1]. In addition, the study of trace elements in the environment has drawn much attention to the presence of critical raw materials (CRMs) like germanium (Ge), rare earth elements (REEs), and potentially toxic elements (PTEs) in different kinds of waste and combustion products. Some of these elements are widely dispersed in soils and do not exist in concentrated deposits [2,3,4,5,6,7].

The environmental presence of these elements of interest has implications that are either negative or positive, depending on their concentration and the sensitivity of the living organisms in the environment. Potentially toxic elements and some CRMs have negative consequences on living organisms when they exist in concentrations that are beyond permissible limits, as has been revealed by some studies [8,9]. Their effect on biochemical reactions in living organisms can impact metabolic processes and reduce crop yields [1]. Thus, there is a need for remediating the environment when these PTEs exist in toxic concentrations. In addition, the presence of CRMs in soils and various depositories such as waste implies that there is the possibility of element recovery via urban mining to increase the supply of CRMs since the economic development of these CRMs, despite the increasing demand and price, has not been sustainable [1,6,7,10,11].

Phytoextraction is among the several techniques that can be used to remediate the high presence of PTEs in soil and biologically extract CRMs (phytoremediation for PTEs and phytomining for CRMs). It is cost effective and has less environmental impact [12]. It involves the use of plants to sequester elements from the soil via the roots [13]. However, phytoextraction can be limited by a low availability of elements in the soil for uptake and low plant biomass production. This is because some elements may not be available in chemical species readily available for plant uptake as they exist in different soil fractions of potentially mobile element forms bound to clays, minerals, and oxides of iron and manganese, which has a strong influence on their behavior in soil and availability for phytoextraction. One example is iron (Fe), which exists as iron hydroxide in soil. The hydroxide is solubilized by bacteria to free the iron ion or the iron is solubilized by siderophore released by some soil bacteria, as reported by Schwabe [14]. These bacteria impact the solubility by changing the speciation of the element of interest in the rhizosphere, hence the plethora of studies that are targeted towards understanding the chemical behavior and bioavailability of these elements of interest in soil and enhancing the process of phytoextracting them from soil [10,13,15,16,17,18].

The improvement of soil health and the bioavailability of elements can be done via bioaugmentation using soil microbes [18]. The bioavailability of elements greatly determines the success and long-term sustainability of phytomining and phytoremediation, implying that bioaugmentation with associated plant growth-promoting rhizobacteria (PGPR) could enhance the efficacy of phytoextraction [19]. Plant growth-promoting rhizobacteria form a kind of beneficial symbiotic association with plants where the plant exudates serve as a carbon source for bacteria [13]. They enhance element mobility and bioavailability through several mechanisms, such as the secretion of chelating agents—such as siderophores, phenolic compounds, and organic acids—as well as inducing the acidification or redox changes in the plant rhizosphere [17]. Thus, they augment the capacity of plants for the remediation of contaminated soil and the reduction of the phytotoxicity of PTEs.

In addition, many studies have reported these PGPR strains as being capable of solubilizing phosphate in soil, including a recent one by Schwabe et al. [14]. However, the strains are outnumbered by other bacteria that are easily established in the rhizosphere such that they cannot compete favorably. This limits the amount of P solubilized and the expression of other beneficial mechanisms through which these bacteria influence element bioavailability and plant growth. Therefore, to maximize the benefit of the plant growth-promoting traits of these bacteria, the inoculation of plants or soil by higher concentrations of bacteria than those usually found in soils is required [20]. Some of these PGPRs have been produced at a commercial scale as microbial formulations are used in agriculture as microbial inoculants in soil bioaugmentation [21].

Several studies have demonstrated the involvement of beneficial micro-organisms, such as rhizobacteria or endophytes associated with plant roots, for the extraction or accumulation of elements of interest or for reducing toxicity and the immobilization of elements in soil [13]. *Pseudomonas maltophilia* was reported to have reduced the toxicity of chromium (Cr) in soils by reducing the toxic Cr^6+^ to nontoxic and immobile Cr^3+^ and to have restricted the mobility of toxic ions like cadmium (Cd^2+^), lead (Pb^2+^), and mercury (Hg^2+^) [13,22,23]. Rajkumar and Freitas [24] also observed that the inoculation of *Ricinus communis* with *Pseudomonas sp*. PsM6 or *P. jessenii* PjM15 increased plant biomass production and enhanced the phytoextraction efficacy for nickel (Ni), copper (Cu), and zinc (Zn) by the production of indole-3-acetic acid (IAA) and solubilizing phosphate. *Bacillus amyloliquefaciens* BSL16 was reported to increase Cu accumulation and the growth of rice seeds and tomato plants under Cu stress [25]. Furthermore, Abou-Shanab et al. [26] reported the possibility of an increase in Ni accumulation by rhizobacteria. *Bacillus lichenformis* was reported to have enhanced the accumulation of Cu, Cd, Pb, and Cr [27]. In addition, a recent study by Kabeer et al. [28] reported a reduced shoot content of Cu and Pb upon treatment with rhizobacteria, while Schwabe et al. [14] reported an increased shoot content of Ge and REEs upon inoculation with PGPR.

These studies have highlighted the roles that PGPR plays in plant element accumulation. However, to the best of our knowledge, the effects of bioaugmentation by *B. amyloliquefaciens* FZB42 inoculated via the commercially available formulation RhizoVital^®^42 on the simultaneous uptake of PTEs, CRMs such as Ge and REEs, nutrients, shoot yield, and bacterial community composition using *Fagopyrum esculentum* cv *Moench* and *Zea mays* cv *Badischer Gelber* as test plants and for the purpose of phytomining and phytoremediation have not been studied. Therefore, the main aim of this study was to evaluate the effects of bioaugmentation using inoculum from a commercially produced microbial formulation of *B. amyloliquefaciens* FZB42 on the phytoextraction of PTEs (arsenic (As), lead (Pb), cobalt (Co), copper (Cu)) and CRMs (germanium (Ge), and the sum total of REEs (REET)), as well as iron (Fe), silicon (Si), calcium (Ca), and phosphorus (P)—regarded as the nutrient elements in this study—from soil. We hypothesized that the inoculation of soil with Rhizovital 42 (bioformulated *B. amyloliquefaciens* FZB42) inoculum will enrich the strain in soil, and improve plant shoot yield and the aboveground phytoaccumulation of elements, given the reports of the effects of PGPR on element accumulation from previous studies.

## 2. Results

### 2.1. Effect of Inoculation on Soil Microbial Community Composition and B. amyloliquefaciens Abundance in Soil

**Figure 1:**
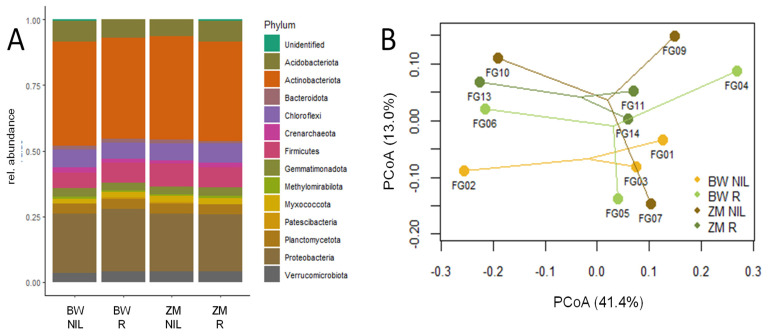
Bacterial community composition in the plant rhizosphere at plant harvest. (**A**) Bar plot showing the average distribution of main phyla (with abundances of >0.5%) in the soils. (**B**) Visualization of a multidimensional scaling approach (PCoA) to explore dissimilarities between the soil communities. The respective three replicates of each color-coded treatment are connected to each other. ZM = maize (*Z. mays*), BW = buckwheat (*F. esculentum*), NIL = reference soil, R = inoculated soil, FGxx = sample ID.

The analyses of the bacterial community at the end of the experiment revealed no significant differences between the studied treatments. Neither the crop nor the application of Rhizovital showed significant effects on the relative abundance of main bacterial phyla (Figure 1A, Table 1) or on the community composition (Figure 1B). At the phylum level, Actinobacteriota predominated all soil communities (with a mean of 28%, Figure 1A, Table 1), followed by Proteobacteria (18.4%), Acidobacteriota (10.1%), Chloroflexi (7.8%), Firmicutes (7.3%), and Planctomycetota (7.2%). Although the principal coordinates analysis (PCoA) indicated dissimilarities between the bacterial communities (Figure 1B), these differences were not related to the applied treatments, indicating that the inoculated strain did not affect the inherent soil community.

Regarding the investigated target strain *Bacillus amyloliquefaciens* FZB42, the results of Illumina sequencing show that compared to reference soils for both plants, soils inoculated with *B. amyloliquefaciens* generated a lower number of sequences (*F. esculentum* = 61,553, *Z. mays* = 50,967) than uninoculated soils (*F. esculentum* = 62,317, *Z. mays* = 55,217) and had a lower number of operational taxonomic units (OTUs) (*F. esculentum* = 1641, *Z. mays* = 1567) than inoculated soils (*F. esculentum* = 1718, *Z. mays* = 1570). In addition, the results show that soils in which *F. esculentum* was grown generated a higher number of sequences and had higher OTU numbers compared to the soils planted with *Z. mays*. For *F. esculentum*, inoculated soils generated 764 and 77 fewer sequences and OTUs, respectively, than uninoculated soils, while for *Z. mays*, soils inoculated with PGPR generated 4250 and 3 fewer sequences and OTUs, respectively, than uninoculated soils. In reference soils in which *F. esculentum* was grown, no sequences related to the inoculated strain were found, whereas in soils inoculated with the PGPR, approximately 510 sequences were generated. Similar observations were found for the reference soils (four sequences generated from just a single replicate) versus inoculated soils (383 sequences generated) in which *Z. mays* was grown. Therefore, the results demonstrate that the strain *B. amyloliquefaciens* was present in the inoculated soils with average relative abundances of 0.85% and 0.75% for the bacterial soil communities of *F. esculentum* and *Z. mays*, respectively.

### 2.2. Effect of PGPR on Shoot Yield and Accumulation of Investigated Elements

**Figure 2:**
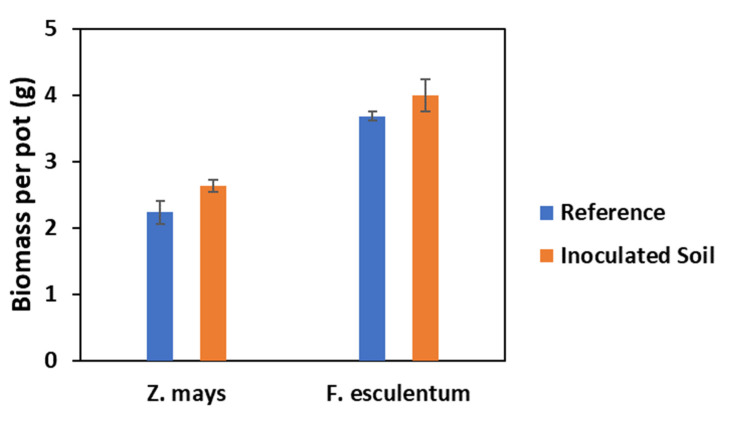
Effect of inoculation on shoot yield of *Zea mays* and *Fagopyrum esculentum* (mean ± SE, *n* = 3).

**Figure 3:**
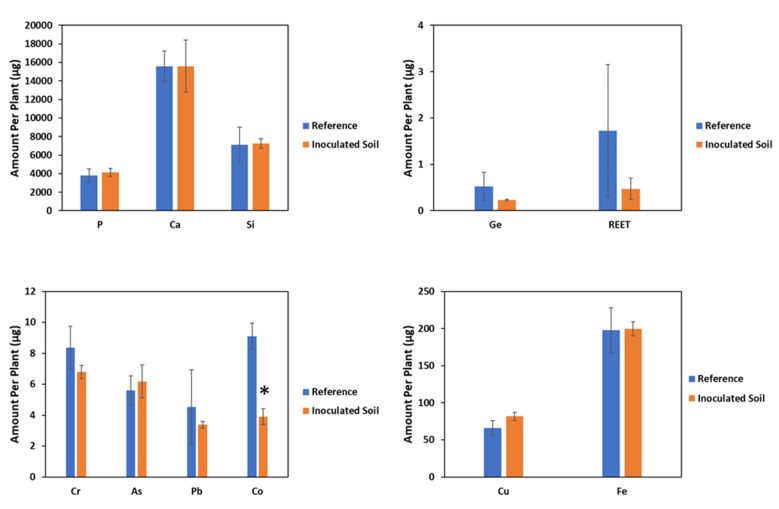
Effect of inoculation on phytoaccumulation of investigated elements by *Zea mays*. Significant difference (*p* ≤ 0.1) between means indicated by asterisk * (mean ± SE, *n* = 3).

For both *Z. mays* and *F. esculentum*, there were no significant differences between the biomass produced by plants grown on reference soils and soils inoculated with *B. amyloliquefaciens*. Inoculation with PGPR only slightly affected the shoot yield of *F. esculentum* and *Z. mays*. Inoculated plants showed an 8% higher shoot yield for *F. esculentum* and an 18% higher yield for *Z. mays* compared to the reference plants (Figure 2). For *Z. mays,* inoculation with *B. amyloliquefaciens* FZB42 did not significantly alter the accumulation of nutrient elements, Ge, REET, and most PTEs considered in this study except Co, for which there was a significant decrease in accumulation of 57% (Figure 3). Contrastingly, the inoculated plants displayed slight increases of 10% and 23% in the shoot contents of Cu and As, respectively.

**Figure 4:**
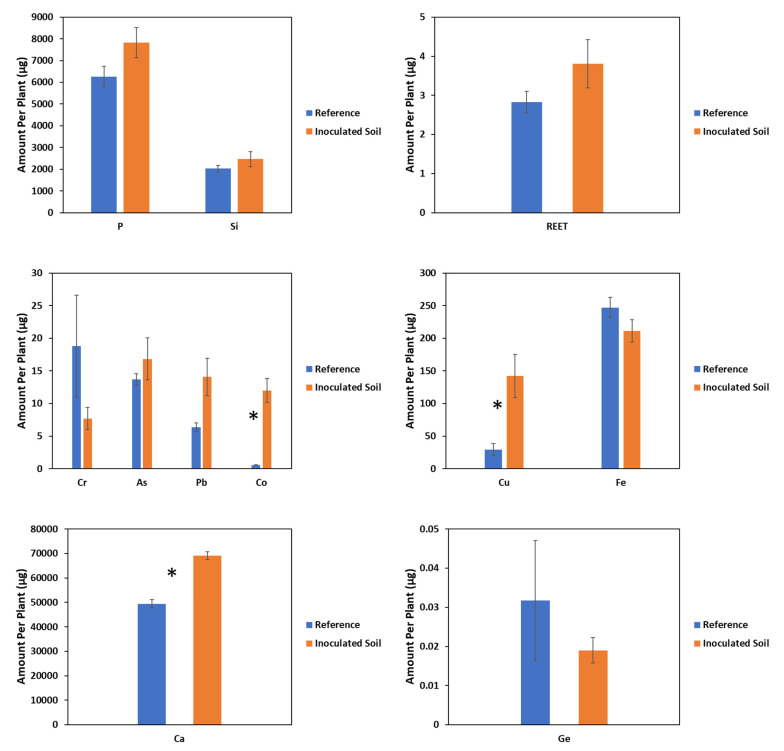
Effect of inoculation on phytoaccumulation of investigated elements by *Fagopyrum esculentum*. Significant difference (*p* ≤ 0.1) between means indicated by asterisk * (mean ± SE, *n* = 3).

In addition, in *Z. mays*, concentrations (Table 2 and Table 3) of the most investigated elements decreased by percentages between 6% and 75%, with the exception of Cu, which was not affected. For *F. esculentum* growing on inoculated soils, the shoot contents of Cr, Fe, and Ge decreased by 59%, 15%, and 40% respectively, while the accumulation of the rest elements was not significantly impacted except for Ca, Cu, and Co, for which there were significant increases of 40%, 383%, and 2042%, respectively (Figure 4). In addition, observations for the effect of inoculation on the concentrations of the investigated elements in *F. esculentum* (Table 2 and Table 3) were similar to the observations for the effects of inoculation on the shoot contents of the investigated elements.

## 3. Discussion

### 3.1. Effects of Inoculation on Root Colonization, Rhizosphere Bacterial Communities, Nutrient Supply, and Plant Growth

Important aspects for the application of PGPR inoculation-assisted plant biomass production and phytoremediation include the establishment of the inoculant in the soil as well as the effect of the inoculant on the existing microbial community. This is important because it has been reported that bacterial communities in soils are often resistant to the introduction of foreign species [29], which could hinder the establishment and effectiveness of the inoculant [30]. In addition, inoculants could be invasive and alter the existing soil microbial community composition [31], although the success of an invasion is dependent on the diversity of the existing microbial community [32]. Thus, we assessed the relative abundance of *B. amyloliquefaciens* in the soil community and checked for differences between the bacterial community composition in the soils. The results of this study, which show that the strain established itself in the soil community with a relative abundance of approximately 1%, indicate a successful integration of the strain into the bacterial community. The high abundance of the inoculated strain in the soil indicates that the existing microbial community did not prevent the establishment of the strain in the soil. This finding could be related to the fact that *Bacillus* species are known to produce endospores that help them survive and establish themselves in soil [27,31]. In addition, a possible restricted niche overlap in the soil between *B. amyloliquefaciens* and the resident bacteria, which is sometimes influenced by a variation in nutrient demands and spatial separation, may have contributed to the establishment of *B. amyloliquefaciens* in the soil. In addition, the results of the PCoA, which show that inoculation did not cause a significant shift in the bacterial community composition, agree with the findings of Chowdhury et al. [33], who reported that *B. amyloliquefaciens* FZB42 did not significantly impact the indigenous rhizosphere bacterial community. Niche processes, which are determined by plant selection power and other environmental factors, such as soil chemistry, are the major factors driving microbial community assemblage in the rhizosphere [34,35,36]. The absence of a significant shift in the microbial community composition suggests that inoculation with *B. amyloliquefaciens* did not impact plant selection power or other environmental factors enough to cause a significant shift in the niche processes within the soil microbial community. This alleviates the fears that the inoculation of soil with *B. amyloliquefaciens* may significantly disturb the structure of the microbial community and the fear that *B. amyloliquefaciens* will not survive in soil when used as an inoculant, confirming that they are safe for use in agriculture and phytoremediation purposes.

### 3.2. Effects of Inoculation on Shoot Yield

In this study, we used fertile PTE-polluted soil from the post-mining area of Freiberg. Thus, it was not surprising that the biomass production (shoot yield) was only slightly affected by inoculation under the conditions of adequate nutrient supply, as evident in the slight increase in the biomass of the inoculated plants compared to the non-inoculated reference plants. This slight increase, although insignificant, could be due to the plant growth-promoting properties of *B. amyloliquefaciens* related to the secretion of indole acetic acid (IAA) and 1-aminocyclopropane-1-carboxylic acid deaminase (ACC deaminase) activity, some of which promote increased photosynthetic rates [37,38,39,40]. Stefan et al. [41] reported increased photosynthetic rates in runner bean upon inoculation with two PGPRs, stating the IAA-producing ability of the bacteria as a possible cause. Similarly, Naveed et al. [42] reported enhanced shoot biomass production and physiology (photosynthesis, chlorophyll content, and efficiency of photosystem II) in *Z. mays* upon inoculation with endophytic PGPR, which colonized the plants. In addition, an increased acquisition of nutrients may have contributed to the slight increase in the biomass observed, but this would be mostly true for *F. esculentum*, where inoculation increased the accumulation of most nutrients (P, Si, and Ca) between 22% and 25% compared to *Z. mays*, where the slight percentage increase upon inoculation was not more than 8%. The increased accumulation of nutrients might be a result of a *B. amyloliquefaciens*-induced increase in the nutrient element solubilization and the mobility of these nutrients in the rhizosphere, thus making these elements bioavailable for plant uptake. A *Bacillus* species was reported by Jamil et al. [43] to have increased Ca and P accumulation in plants, and this is in tandem with the results of our study. The reduced accumulation of Fe, despite *B. amyloliquefaciens* being a siderophore-producing bacterium, may be because the siderophore produced under the conditions in the substrate favored the solubility and binding of metals other than Fe, hence the decrease in the accumulation of Fe [44].

### 3.3. Effects of Inoculation on PTE and CRM Accumulation

The effect of *B. amyloliquefaciens* on plant growth is of interest for plant growth promotion in agriculture and biomass production for bioenergy purposes, especially on marginal soils characterized by high concentrations of PTEs. However, beyond these reasons, there is interest in the effects of *B. amyloliquefaciens* on the phytoextraction of elements from soil, for example, PTEs [45] and CRMs such as Ge and REEs.

In this study, the observed effects of inoculation on element accumulation by *F. esculentum* (a forb and strategy 1 plant with respect to Fe acquisition) and *Z. mays* (a grass and strategy 2 plant with respect to Fe acquisition) differed for some elements and were similar for others. These differences in the observed effects may be related to the plant species’ characteristics, such as growth habits, element acquisition strategy, and colonization of the plant roots by bacteria [17]. In addition, although the effects of many elements on accumulation by both test plants upon inoculation were substantial, these effects were statistically insignificant for most elements, possibly due to variation in the extent of inoculation effects among plant replicates. Plants were placed in a randomized manner under the light source, causing differences in intensity of light exposure among replicates. These differences can affect the photosynthetic and transpiration rates among plant replicates, which could have an effect on the extent inoculation affects plant replicates. Only the effects of inoculation on Ca and Cu phytoextracted by *F. esculentum* and Co phytoextracted by both test plants were significant. The increased accumulation of Cu and As in *Z. mays*, as well as Cu, As, Co, and REET in *F. esculentum* upon inoculation with *B. amyloliquefaciens* may be connected with the solubilization of these elements by substances produced by the bacteria, such as carboxylic acids, indole acetic acids, and siderophores, as well as root exudates produced by plants, which solubilize these metals and facilitate their uptake by the plant roots [13]. The formation of siderophore–metal complexes and the release of elements from organic matter decomposition by bacteria, which can be taken up directly by plants, increases the accumulation of metals in plants [17,46]. These results agree with those of Khan et al. [25], who reported that *Bacillus amyloliquefaciens* BSL16 increased the accumulation of Cu in rice and stated the production of organic acids, biosurfactants, and siderophores as possible reasons for the increased Cu accumulation, as suggested by Sheng et al. [47]. Additionally in agreement with our results are those from the study of Lampis et al. [48], who reported a 22% increase in As accumulation upon plant inoculation with PGPR, crediting the increase to the combined effect of the beneficial properties of siderophore and IAA production by the PGPR, as well as the reduction of arsenate to arsenite.

The contrasting results of the decreased accumulations of Cr, Pb, Co, Ge, and REET in *Z. mays*, as well as of Cr and Ge in *F. esculentum* may be due to a possible immobilization of these elements in the soil upon inoculation with bacteria, thus limiting uptake by *Z. mays*. It is possible that *B. amyloliquefaciens* used polymeric substances, exopolysaccharides that are capable of forming biofilms around plant roots, and other chemical substances, such as some carboxylates it produces to immobilize these elements by forming stable complexes with their ions in the soil solution, thus limiting their uptake by plants [27,49,50,51]. Ashraf et al. [52] reported the formation of soil sheaths in the root zone of wheat to limit the flow of toxic ions into wheat roots upon inoculation with exopolysaccharide producing *Bacillus* spp. Fan et al. [53] reported that the expression of genes involved in the formation of biofilms was enhanced by maize root exudates. Silva et al. [54] reported that the inoculation of *Z. mays* with some PGPR strains reduced the accumulation of Cr in *Z. mays*, and this reduction in the accumulation of Cr may be due to the reduction of the mobile Cr^6+^ to the immobile toxic Cr^3+^ ions, as reported by Jing et al. [13]. This agrees with the results of our study and suggests that reductions in the oxidation states of element ions in the soil, which lead to element immobilization and reduced bioavailability, might be the reason for the reduced uptake of some elements upon inoculation with PGPR. However, some studies have reported a decrease in As accumulation in plants upon inoculation with PGPR, including *Bacillus* [51,55].

Furthermore, element accumulation patterns upon inoculation may have been due to chemical relationships or similarities in origin that resulted in simultaneous accumulation by plants, as the plant may not have easily taken them up differentially or, in some cases, because of competition for the same transport channels or sites. For example, the observed higher accumulation of As and P in *Z. mays* upon inoculation may be connected to the chemical relationship between As and P [56]. In addition, Ge and Cr are usually bound to silicates [6,57,58] and, as such, it may be that the increased accumulation of Si was a result of preferential accumulation of Si over Ge and Cr. Other examples could be Pb and P [59], P and Ca [60], Ca and REET [61].

Conclusively, our study has highlighted the possibilities of enhanced biomass production and phytoextraction of elements, including nutrients, PTEs, and elements of economic value, using *Z. mays* and *F. esculentum* as test plants and commercially available *B. amyloliquefaciens* FZB42 bioformulation as the inoculant. We demonstrated that it is possible that upon inoculation of soil with bacteria, biomass production by *Z. mays* and *F. esculentum* can be enhanced, while phytoextraction can be enhanced or impeded depending on several interacting factors related to plant species characteristics, such as growth habits, element acquisition strategy, and the colonization of plants by bacteria, which could differ between the two plant species [17]. In addition, the study highlights that the use of commercially available microbial inoculant containing *B. amyloliquefaciens* FZB42 as the PGPR, as well as for phytoremediation purposes, is safe, as the *B. amyloliquefaciens* FZB42 establishes itself well in soil and does not majorly affect the structure of the indigenous soil microbial composition. Although the above-mentioned effects of inoculation might not all be significant, we think that they are meaningful, as they indicate what possibilities of element accumulation there could be upon the inoculation of soils in which *F. esculentum* and *Z. mays* are grown, using *B. amyloliquefaciens* as the microbial inoculant. Thus, the findings of this study may provide useful information when planning agricultural projects that intend to use microbes to boost plant growth and nutrient content, for environmental remediation projects that intend to use plants and microbes to enhance the extraction of economically valuable elements and contaminants from soil, and for biomass for bioenergy projects that intend to use microbes to enhance plant biomass production.

## 4. Materials and Methods

### 4.1. Plant Growth Experiment and Soil Amendment

The plant species used as test plants in this study were *Zea mays* cv *Badischer Gelber* and *Fagopyrum esculentum* cv *Moench*, which were grown under constant laboratory conditions of a temperature of 25 °C and light exposure time of 12 h per day. The plants were grown in 3 replicates, each in 2 kg of potted soils obtained from the vicinity of Technische Universität Bergakademie Freiberg, which represent typical soils of the Freiberg area of Germany [62]. Five seeds of each plant species were initially sown per pot but reduced to one plant per pot after 2 weeks post-germination. Plants grown in non-inoculated soil served as the reference for those grown in soils inoculated with *Bacillus amyloliquefaciens*. An inoculation rate of approximately 0.4% (0.4 mL of inoculum in 100 mL) per pot was used, and the soil was inoculated twice (100 mL of 0.4% inoculum mixture each time) within the 53-day growing period of the experiment, with a time interval of 2 weeks between inoculations. Rhizovital 42 (bioformulated *Bacillus amyloliquefaciens*), supplied by ABiTEP GmBH Berlin and containing 2.5 × 10^10^ CFU/mL (colony-forming units per milliliter) of *Bacillus amyloliquefaciens*, was the source of inoculum.

### 4.2. Sample Preparation and Analysis

#### 4.2.1. Soil Samples (Before Inoculation)

According to Du Laing [63], readily available element fractions include the mobile/exchangeable and acid-soluble element pools. The concentrations of the elements in these fractions were determined via sequential extraction according to the methods described by Wiche and Heilmeier [6]. To determine the total element concentrations, 10 portions of the soil samples were dried at 105 °C and ground in a boron carbide mortar. Then, 0.5 g of the ground soil and 2 g of an equivalent mixture of Na_2_CO_3_ and K_2_CO_3_ were placed in a nickel crucible and thoroughly mixed for melting digestion, according to the methods by Alfassi and Wai [64]. The mixture was heated in a muffle furnace for 30 min at 900 °C, after which the samples were cooled and dissolved with 50 mL of a 2 M nitric acid and 0.5 M citric acid solution. The resulting solutions from the melting digestion and sequential extraction were diluted, and the concentrations of the elements were determined using ICP-MS (X series 2, Thermo Fisher Scientific, Dreieich, Germany). The accuracy of the analytical process was checked using certified reference material (NCS ZC73032 and NCS ZC73030) [65]. The results deviated by less than 10% from the certified values.

The physico-chemical properties of the uninoculated soil, the concentrations of the readily available soil element fractions, and the total element concentrations are reported in Table 4. Soil electrical conductivity was 32 µS/cm, while the soil organic matter content, determined by the loss of ignition, was 7.7 %. The soil pH was 6.2 and in the effective range for soil microbial functions and nutrient availability but not for the bioavailability of most of the CRMs considered in this study [66,67]. The total concentrations of Ge and REEs were similar to those reported by Wiche et al. [62], with the total concentration of PTEs more than the threshold values allowed for European soils, as reported by Tóth et al. [68], which is due to previous mining activities in the region of Freiberg. Of the readily available PTEs, Pb had the highest concentration (36.6 µg/g), while the concentrations of readily available As, Cu, Co and Cr, and Co were 1.13 µg/g, 1.53 µg/g, 0.34 µg/g, and 0.34 µg/g, respectively. The readily available concentrations of the sum total of REEs (3.79 µg/g) were quite higher than that of Ge (0.02 µg/g). For the selected nutrients, the concentrations of the readily available fractions were P (58.9 µg/g), Fe (23.5 µg/g), Ca (2514 µg/g), and Si (117 µg/g). These concentrations mean that the soil was polluted but not nutrient deficient or infertile.

#### 4.2.2. Plant Samples

During harvest, the plants were cut off at heights between 2–3 cm above ground level, weighed, and dried at 60 °C in an oven (model SIM 500, Memmert, Schwabach, Germany) for 48 h to obtain a constant weight. Subsequently, the dry mass of the samples was determined and pulverized to a fine powder using an ultra-centrifugal mill (model ZM1000, Retsch, Haan, Germany). Then, 100 mg of the dried pulverized plant samples were weighed out for digestion in a microwave (MLS-ETHOS plus, MLS GmbH, Dorsten, Germany) according to the methods by Krachler et al. [69]. Before digestion, the samples were mixed with 200 µL of ultra-pure water as well as with 1.9 mL nitric acid and left overnight to react before adding 600 µL of 4.9% hydrofluoric acid. After digestion, the samples were transferred into 15 mL centrifuge tubes, with volumes of up to 10 mL. For the measurement of trace elements, Ge, and REEs using ICP-MS (model X Series 2, Thermo Fisher Scientific, Dreieich, Germany), 1 mL each from the diluted samples were further transferred to 15 mL Teflon tubes before adding 100 µL of internal standards containing 1 mg/L of rhodium and rhenium, according to the methods by Krachler et al. [69], with volumes of up to 10 mL. The accuracy of the analytical process was checked using certified reference material (NCS ZC73032 and NCS ZC73030) [62]. The results deviate by less than 10% from the certified values.

#### 4.2.3. Soil DNA Extraction and Illumina Sequencing

Microbial DNA was extracted from approximately 250 mg soil, which had been collected immediately after plant harvest and preserved at −80 °C. The extraction procedure was done using a QIAGEN DNeasy Power Soil kit and based on the specifications of the manufacturer. Before storing the DNA extracts at −20 °C, the DNA concentrations in the extracts were examined with a NanoDrop ND-8000 spectrophotometer (Thermo Fischer Scientific, Dreieich, Germany). For the PCR, the DNA concentrations of the extracts were adjusted to 10–15 ng/µL. Amplification of the bacterial 16S rRNA gene V4 region was performed in triplicate for each sample with the universal primers 515f and 806r [70], which were equipped with Illumina adapter sequences. To ensure the correct amplification of the sequences, proofreading KAPA HiFi polymerase was used for all PCR reactions (KAPA Biosystems, Boston, MA, United States). The PCR reaction consisted of 7.5 µL of KAPA polymerase, 0.3 µL of each primer (10 µM), 5.9 µL of water, and 1 µL of DNA template, and was conducted with the PCR conditions summarized in Table 5 (PCR1). The PCR products were checked by gel electrophoresis, and triplicates for each sample were pooled together. After purification of the PCR products with the Agencourt AMPure XP kit (Beckmann Coulter, Krefeld, Germany), Illumina Nextera XT indices were attached to both ends of the bacterial fragments in a second PCR (PCR2, Table 5) in order to assign the sequences to the respective samples. The PCR products were purified using AMPure beads, and the DNA was quantified with the PicoGreen assay (Molecular Probes, Eugene, OR, United States). For an equimolar representation of each sample, defined volumes of the prepared bacterial amplicon libraries were pooled together. The fragment size and the quality of the final DNA sequencing library pool were again checked with the Agilent 2100 Bioanalyzer (Agilent Technologies, Palo Alto, CA, United States). Finally, paired-end sequencing of 2 × 300 bp was implemented on an Illumina MiSeq platform (Illumina Inc., San Diego, CA, United States) at the Department of Soil Ecology of the Helmholtz Centre for Environmental Research (UFZ, Halle/Saale, Germany).

#### 4.2.4. Bioinformatics Workflow

Demultiplexed sequences were processed using the “dadasnake” pipeline [71], which is based on the implementation of the DADA2 package [72] from the open-source program R (v. 3.6.1; R Core Team 2017) into Snakemake [73]. 16S rDNA amplicon reads were cut and filtered using the default settings of the pipeline. Read pairs were merged with a minimum overlap of 12 bp and zero mismatches, and chimeric reads were removed using the consensus algorithm. For taxonomical classification of the 16S rDNA gene amplicon sequences, the Mothur implementation of the Bayesian Classifier (Schloss et al. [74]) and—as a follow up in the case of a missing classification—BLASTn were applied, referring to the SILVA database (version 132, non-redundant at 99%; [75]). The final output was comprised of an OTU table with the taxonomic classifications for all samples.

#### 4.2.5. Statistical Analysis

The statistical differences between the treatments for each plant species for shoot contents (amount accumulated), element concentrations, and shoot yield were evaluated using Welch’s analysis of variance (ANOVA) at a significance level of *p* < 0.1 using IBM SPSS Statistics 26 software. Significant differences (*p* ≤ 0.1) between the means indicated are indicated by an asterisk * in the figures. The bar plots and PCoA were created with R, version 4.0.5, using the “vegan” and “ggplot2” packages.

## Figures and Tables

**Table 1:** Mean proportions (given in % of the total community) of main phyla (with abundances of >0.5%) in the soils of the studied treatments. Soils were cultivated with *Fagopyrum esculentum*/buckwheat (BW) or *Zea mays* (ZM) without inoculation (NIL) and with inoculation (R) of *B. amyloliquefaciens*.

Phylum	BW NIL	BW R	ZM NIL	ZM R
Acidobacteriota	10.31	9.81	9.83	10.53
Actinobacteriota	28.98	27.88	27.62	27.39
Bacteroidota	2.83	3.08	2.57	2.21
Chloroflexi	7.97	7.56	7.65	8.00
Crenarchaeota	0.59	0.58	0.61	0.63
Firmicutes	6.67	7.55	7.69	7.09
Gemmatimonadota	4.03	4.31	4.35	4.57
Methylomirabilota	0.74	0.50	0.66	0.74
Myxococcota	3.11	3.33	3.72	3.94
Patescibacteria	1.39	1.66	1.61	1.67
Planctomycetota	7.26	7.58	7.14	6.95
Proteobacteria	18.40	18.37	18.73	18.05
Verrucomicrobiota	2.74	2.65	2.64	2.89
Unidentified	0.72	0.81	0.64	0.79

**Table 2:** Effect of soil inoculation on concentration (µg/g) of PTEs, Ge, and REET in shoots of test plant species.

Species	Treatment	Cr	As	Pb	Co	Cu	Ge	REET
***Z. mays***	**NIL**	3.86 ± 0.90	2.50 ± 0.31	1.93 ± 0.89	4.14 ± 0.51	30.1 ± 5.74	0.26 ± 0.16	0.68 ± 0.54
	**R**	2.58 ± 0.11	2.34 ± 0.38	1.28 ± 0.05	1.48 ± 0.18	31 ± 1.52	0.09 ± 0.004	0.17 ± 0.08
	**Statistic ^a^**	1.97	0.10	0.52	24.0	0.019	1.05	0.87
	***p* value**	0.29	0.77	0.55	0.03	0.9	0.41	0.45
***F. esculentum***	**NIL**	5.15 ± 2.22	3.72 ± 0.18	1.72 ± 0.16	0.15 ± 0.01	7.94 ± 2.49	0.01 ± 0.004	0.77 ± 0.06
	**R**	1.89 ± 0.34	4.14 ± 0.58	3.49 ± 0.58	2.97 ± 0.30	36.1± 8.90	0.005 ± 0.001	0.96 ± 0.17
	**Statistic ^a^**	2.11	0.47	8.68	90.98	9.25	0.81	1.21
	***p* value**	0.28	0.55	0.08	0.011	0.078	0.46	0.37

Mean ± SE, *n* = 3, NIL = reference, R = inoculated soil. Statistic ^a^ means asymptotically distributed F statistic for Welch’s ANOVA.

**Table 3:** Effect of soil inoculation on concentration (µg/g) of selected nutrients in shoots of test plant species.

Species	Treatment	P	Ca	Si	Fe
***Z. mays***	**NIL**	1681 ± 181	6981 ± 611	3137 ± 636	88 ± 8
	**R**	1578 ± 208	5975 ± 1162	2744 ± 142	76 ± 6
	**Statistic ^a^**	0.14	0.59	0.36	1.28
	***p* value**	0.728	0.499	0.603	0.327
***F. esculentum***	**NIL**	1699 ± 122	13,434 ± 692	549 ± 34	67 ± 4
	**R**	1953 ± 94	17,421 ± 1294	611 ± 53	53 ± 4
	**Statistic ^a^**	2.73	7.39	0.95	6.06
	***p* value**	0.18	0.07	0.39	0.07

Mean ± SE, ***n*** = 3, **NIL** = reference, **R** = inoculated soil. Statistic ^a^ means asymptotically distributed F statistic for Welch’s ANOVA.

**Table 4:** Soil physico-chemical parameters and concentrations of elements.

**4a: Soil Physico-Chemical Parameters**			
Water content (*w/w*)	17.9%		
pH value in aqueous solution	6.2		
Conductivity	32.3 µS/cm		
Organic matter content	7.7%		
Nitrate concentration	147 mg/kg		
Ammonium concentration	0.88 mg/kg		
Phosphate concentration	136 mg/kg		
Cation exchange capacity	9.1 cmol/kg		
**4b: Total Concentration and Concentration in Operationally Defined Fractions (µg/g) (mean ± SE)**			
	Total concentration	Fraction 1	Fraction 2
Cu	175 ± 36	0.69 ± 0.04	0.84 ± 0.1
Pb	180 ± 41	5.6 ± 0.8	31 ± 3.2
Cr	111 ± 11	0.10 ± 0.02	0.23 ± 0.01
As	93 ± 25	0.39 ± 0.2	0.73 ± 0.2
Ge	1.84 ± 0.04	0.004 ± 0.001	0.014 ± 0.001
REET	157 ± 3.1	0.99 ± 0.1	2.80 ± 0.2
Ca	5875 ± 675	2282 ± 495	232 ± 45
P	1986 ± 89	33.3 ± 6.3	25.6 ± 8.3
Fe	29,337 ± 551	4.1 ± 0.4	19.4 ± 2.2
Co	24.3 ± 2.1	0.09 ± 0.01	0.24 ± 0.02
Si	141,455 ± 18,019	62.7 ± 9.6	54.7 ± 5.0

Fraction 1 = mobile/exchangeable element fraction, Fraction 2 = acid soluble element fraction. Values are means of 10 replicates except for P (total concentration), whose value is the mean of 7 replicates. Elements in bold letters have concentrations higher than permitted for European soils, as reported by Tóth et al. [66].

**Table 5:** PCR conditions used for next-generation sequencing with Illumina for initial amplification of 16S rRNA gene region (PCR 1) as well as for the index PCR (PCR 2).

	Step	Temperature (°C)	Time (min:sec)
**PCR 1**			
	Initial denaturation	95	**3:00**
**25 cycles**	Denaturation	98	**0:20**
	Annealing	55	**0:15**
	Elongation	72	**0:15**
	Final extension	72	**5:00**
**PCR 2**			
	Initial denaturation	95	**3:00**
**8 cycles**	Denaturation	98	**0:30**
	Annealing	55	**0:30**
	Elongation	72	**0:30**
	**Final extension**	**72**	**5:00**

## Data Availability

The data presented in this study are available on request from the corresponding author. The data are not publicly available yet because they are yet to be put in an online repository.

## References

[1] Turan M., Topcuoğlu B., Kitir N., Alkaya Ü., Erçelik F., Nikerel E., Günes A., Larramendy M.L., Soloneski S. (2016). Plant Growth Promoting Rhizobacteria’s (PGPRS) Enzyme Dynamics in Soil Remediation. Soil Contamination-Current Consequences and Further Solutions.

[2] Zhang F.-S., Yamasaki S., Kimura K. (2001). Rare Earth Element Content in Various Waste Ashes and the Potential Risk to Japanese Soils. Environ. Int..

[3] Zhao C., Duan D., Li Y., Zhang J. (2012). Rare Earth Elements in No. 2 Coal of Huangling Mine, Huanglong Coalfield, China. Energy Explor. Exploit..

[4] Kaartinen T., Sormunen K., Rintala J. (2013). Case Study on Sampling, Processing and Characterization of Landfilled Municipal Solid Waste in the View of Landfill Mining. J. Clean. Prod..

[5] Gutiérrez-Gutiérrez S.C., Coulon F., Jiang Y., Wagland S. (2015). Rare Earth Elements and Critical Metal Content of Extracted Landfilled Material and Potential Recovery Opportunities. Waste Manag..

[6] Wiche O., Heilmeier H. (2016). Germanium (Ge) and Rare Earth Element (REE) Accumulation in Selected Energy Crops Cultivated on Two Different Soils. Miner. Eng..

[7] Hussain R., Luo K. (2020). Geochemical Evaluation of Enrichment of Rare-Earth and Critical Elements in Coal Wastes from Jurassic and Permo-Carboniferous Coals in Ordos Basin, China. Nat. Resour. Res..

[8] Adeel M., Shakoor N., Hussain T., Azeem I., Zhou P., Zhang P., Hao Y., Rinklebe J., Rui Y. (2021). Bio-Interaction of Nano and Bulk Lanthanum and Ytterbium Oxides in Soil System: Biochemical, Genetic, and Histopathological Effects on Eisenia Fetida. J. Hazard. Mater..

[9] Adeel M., Shakoor N., Ahmad M.A., White J.C., Jilani G., Rui Y. (2021). Bioavailability and Toxicity of Nanoscale/Bulk Rare Earth Oxides in Soil: Physiological and Ultrastructural Alterations in *Eisenia fetida*. Environ. Sci. Nano.

[10] El-Ramady H. (2010). Ecotoxicology of Rare Earth Elements: Ecotoxicology of Rare Earth Elements within Soil and Plant Environments.

[11] Franus W., Wiatros-Motyka M.M., Wdowin M. (2015). Coal Fly Ash as a Resource for Rare Earth Elements. Environ. Sci. Pollut. Res..

[12] Kasowska D., Gediga K., Spiak Z. (2018). Heavy Metal and Nutrient Uptake in Plants Colonizing Post-Flotation Copper Tailings. Environ. Sci. Pollut. Res..

[13] Jing Y., He Z., Yang X. (2007). Role of Soil Rhizobacteria in Phytoremediation of Heavy Metal Contaminated Soils. J. Zhejiang Univ. Sci. B.

[14] Schwabe R., Dittrich C., Kadner J., Rudi Senges C.H., Bandow J.E., Tischler D., Schlömann M., Levicán G., Wiche O. (2021). Secondary Metabolites Released by the Rhizosphere Bacteria Arthrobacter Oxydans and Kocuria Rosea Enhance Plant Availability and Soil–Plant Transfer of Germanium (Ge) and Rare Earth Elements (REEs). Chemosphere.

[15] Li F., Shan X., Zhang T., Zhang S. (1998). Evaluation of Plant Availability of Rare Earth Elements in Soils by Chemical Fractionation and Multiple Regression Analysis. Environ. Pollut..

[16] Violante A., Cozzolino V., Perelomov L., Caporale A.G., Pigna M. (2010). Mobility and bioavailability of heavy metals and metalloids in soil environments. J. Soil Sci. Plant Nutr..

[17] Benizri E., Kidd P.S., Van der Ent A., Echevarria G., Baker A.J.M., Morel J.L. (2018). The Role of the Rhizosphere and Microbes Associated with Hyperaccumulator Plants in Metal Accumulation. Agromining: Farming for Metals.

[18] Ku Y., Rehman H.M., Lam H.M. (2019). Possible Roles of Rhizospheric and Endophytic Microbes to Provide a Safe and Affordable Means of Crop Biofortification. Agronomy.

[19] Kidd P., Barceló J., Bernal M.P., Navari-Izzo F., Poschenrieder C., Shilev S., Clemente R., Monterroso C. (2009). Trace Element Behaviour at the Root–Soil Interface: Implications in Phytoremediation. Environ. Exp. Bot..

[20] Rodríguez H., Fraga R. (1999). Phosphate Solubilizing Bacteria and Their Role in Plant Growth Promotion. Biotechnol. Adv..

[21] Parray J.A., Jan S., Kamili A.N., Qadri R.A., Egamberdieva D., Ahmad P. (2016). Current Perspectives on Plant Growth-Promoting Rhizobacteria. J. Plant Growth Regul..

[22] Blake R.C., Choate D.M., Bardhan S., Revis N., Barton L.L., Zocco T.G. (1993). Chemical Transformation of Toxic Metals by a *Pseudomonas* Strain from a Toxic Waste Site. Environ. Toxicol. Chem..

[23] Park C.H., Keyhan M., Wielinga B., Fendorf S., Matin A. (2000). Purification to Homogeneity and Characterization of a Novel *Pseudomonas Putida* Chromate Reductase. Appl. Environ. Microbiol..

[24] Rajkumar M., Freitas H. (2008). Influence of Metal Resistant-Plant Growth-Promoting Bacteria on the Growth of Ricinus Communis in Soil Contaminated with Heavy Metals. Chemosphere.

[25] Khan A.L., Bilal S., Halo B.A., Al-Harrasi A., Khan A.R., Waqas M., Al-Thani G.S., Al-Amri I., Al-Rawahi A., Lee I.-J. (2017). *Bacillus amyloliquefaciens* BSL16 Improves Phytoremediation Potential of *Solanum Lycopersicum* during Copper Stress. J. Plant Interact..

[26] Aboushanab R., Angle J., Chaney R. (2006). Bacterial Inoculants Affecting Nickel Uptake by Alyssum Murale from Low, Moderate and High Ni Soils. Soil Biol. Biochem..

[27] Radhakrishnan R., Hashem A., Abd_Allah E.F. (2017). Bacillus: A Biological Tool for Crop Improvement through Bio-Molecular Changes in Adverse Environments. Front. Physiol..

[28] Kabeer R., Sylas V.P., Praveen Kumar C.S., Thomas A.P., Shanthiprabha V., Radhakrishnan E.K., Baiju K.R. (2021). Role of Heavy Metal Tolerant Rhizosphere Bacteria in the Phytoremediation of Cu and Pb Using *Eichhornia Crassipes* (Mart.) Solms. Int. J. Phytoremediation.

[29] Björklöf K., Sen R., Jørgensen K. (2003). Maintenance and Impacts of an Inoculated Mer/Luc-Tagged Pseudomonas Fluorescens on Microbial Communities in Birch Rhizospheres Developed on Humus and Peat. Microb. Ecol..

[30] Castro-Sowinski S., Herschkovitz Y., Okon Y., Jurkevitch E. (2007). Effects of Inoculation with Plant Growth-Promoting Rhizobacteria on Resident Rhizosphere Microorganisms. FEMS Microbiol. Lett..

[31] Ambrosini A., de Souza R., Passaglia L.M.P. (2016). Ecological Role of Bacterial Inoculants and Their Potential Impact on Soil Microbial Diversity. Plant Soil.

[32] Litchman E. (2010). Invisible Invaders: Non-Pathogenic Invasive Microbes in Aquatic and Terrestrial Ecosystems: Invasive Microbes. Ecol. Lett..

[33] Chowdhury S.P., Dietel K., Rändler M., Schmid M., Junge H., Borriss R., Hartmann A., Grosch R. (2013). Effects of *Bacillus amyloliquefaciens* FZB42 on Lettuce Growth and Health under Pathogen Pressure and Its Impact on the Rhizosphere Bacterial Community. PLoS ONE.

[34] Lladó S., López-Mondéjar R., Baldrian P. (2018). Drivers of Microbial Community Structure in Forest Soils. Appl. Microbiol. Biotechnol..

[35] Li L., Ma J., Mark Ibekwe A., Wang Q., Yang C.-H. (2018). Influence of Bacillus Subtilis B068150 on Cucumber Rhizosphere Microbial Composition as a Plant Protective Agent. Plant Soil.

[36] Saad M.M., Eida A.A., Hirt H. (2020). Tailoring Plant-Associated Microbial Inoculants in Agriculture: A Roadmap for Successful Application. J. Exp. Bot..

[37] Redondo-Gómez S., Mesa-Marín J., Pérez-Romero J.A., López-Jurado J., García-López J.V., Mariscal V., Molina-Heredia F.P., Pajuelo E., Rodríguez-Llorente I.D., Flowers T.J. (2021). Consortia of Plant-Growth-Promoting Rhizobacteria Isolated from Halophytes Improve Response of Eight Crops to Soil Salinization and Climate Change Conditions. Agronomy.

[38] Samaniego-Gámez B.Y., Garruña R., Tun-Suárez J.M., Kantun-Can J., Reyes-Ramírez A., Cervantes-Díaz L. (2016). Bacillus Spp. Inoculation Improves Photosystem II Efficiency and Enhances Photosynthesis in Pepper Plants. Chil. J. Agric. Res..

[39] Osorio S., Ruan Y.-L., Fernie A.R. (2014). An Update on Source-to-Sink Carbon Partitioning in Tomato. Front. Plant Sci..

[40] Enebe M.C., Babalola O.O. (2018). The Influence of Plant Growth-Promoting Rhizobacteria in Plant Tolerance to Abiotic Stress: A Survival Strategy. Appl. Microbiol. Biotechnol..

[41] Stefan M., Munteanu N., Stoleru V., Mihasan M., Hritcu L. (2013). Seed Inoculation with Plant Growth Promoting Rhizobacteria Enhances Photosynthesis and Yield of Runner Bean (*Phaseolus coccineus* L.). Sci. Hortic..

[42] Naveed M., Mitter B., Reichenauer T.G., Wieczorek K., Sessitsch A. (2014). Increased Drought Stress Resilience of Maize through Endophytic Colonization by Burkholderia Phytofirmans PsJN and Enterobacter Sp. FD17. Environ. Exp. Bot..

[43] Jamil M., Zeb S., Anees M., Roohi A., Ahmed I., ur Rehman S., Rha E. (2014). shik Role of *Bacillus Licheniformis* in Phytoremediation of Nickel Contaminated Soil Cultivated with Rice. Int. J. Phytoremediation.

[44] Brantley S.L., Liermann L., Bau M. (2001). Uptake of Trace Metals and Rare Earth Elements from Hornblende by a Soil Bacterium. Geomicrobiol. J..

[45] Zhuang X., Chen J., Shim H., Bai Z. (2007). New Advances in Plant Growth-Promoting Rhizobacteria for Bioremediation. Environ. Int..

[46] Ahemad M. (2015). Enhancing Phytoremediation of Chromium-Stressed Soils through Plant-Growth-Promoting Bacteria. J. Genet. Eng. Biotechnol..

[47] Sheng X.-F., Xia J.-J., Jiang C.-Y., He L.-Y., Qian M. (2008). Characterization of Heavy Metal-Resistant Endophytic Bacteria from Rape (Brassica Napus) Roots and Their Potential in Promoting the Growth and Lead Accumulation of Rape. Environ. Pollut. Barking Essex 1987.

[48] Lampis S., Santi C., Ciurli A., Andreolli M., Vallini G. (2015). Promotion of Arsenic Phytoextraction Efficiency in the Fern Pteris Vittata by the Inoculation of As-Resistant Bacteria: A Soil Bioremediation Perspective. Front. Plant Sci..

[49] Qurashi A.W., Sabri A.N. (2012). Bacterial Exopolysaccharide and Biofilm Formation Stimulate Chickpea Growth and Soil Aggregation under Salt Stress. Braz. J. Microbiol. Publ. Braz. Soc. Microbiol..

[50] Fashola M., Ngole-Jeme V., Babalola O. (2015). Diversity of Acidophilic Bacteria and Archaea and Their Roles in Bioremediation of Acid Mine Drainage. Br. Microbiol. Res. J..

[51] Seshadri B., Bolan N.S., Naidu R. (2015). Rhizosphere-Induced Heavy Metal(Loid) Transformation in Relation to Bioavailability and Remediation. J. Soil Sci. Plant Nutr..

[52] Ashraf M.A., Hussain I., Rasheed R., Iqbal M., Riaz M., Arif M.S. (2017). Advances in Microbe-Assisted Reclamation of Heavy Metal Contaminated Soils over the Last Decade: A Review. J. Environ. Manage..

[53] Fan B., Carvalhais L.C., Becker A., Fedoseyenko D., von Wirén N., Borriss R. (2012). Transcriptomic Profiling of *Bacillus amyloliquefaciens* FZB42 in Response to Maize Root Exudates. BMC Microbiol..

[54] Silva R.S., Antunes J.E.L., de Aquino J.P.A., de Sousa R.S., de Melo W.J., Araujo A.S.F. (2021). Plant Growth-Promoting Rhizobacteria Effect on Maize Growth and Microbial Biomass in a Chromium-Contaminated Soil. Bragantia.

[55] Mallick I., Bhattacharyya C., Mukherji S., Dey D., Sarkar S.C., Mukhopadhyay U.K., Ghosh A. (2018). Effective Rhizoinoculation and Biofilm Formation by Arsenic Immobilizing Halophilic Plant Growth Promoting Bacteria (PGPB) Isolated from Mangrove Rhizosphere: A Step towards Arsenic Rhizoremediation. Sci. Total Environ..

[56] Ghosh P., Rathinasabapathi B., Ma L.Q. (2011). Arsenic-Resistant Bacteria Solubilized Arsenic in the Growth Media and Increased Growth of Arsenic Hyperaccumulator *Pteris vittata* L. Bioresour. Technol..

[57] Liu Y., Liu G., Qi C., Cheng S., Sun R. (2016). Chemical Speciation and Combustion Behavior of Chromium (Cr) and Vanadium (V) in Coals. Fuel.

[58] Baleizão C., Gigante B., Sabater M.J., Garcia H., Corma A. (2002). On the Activity of Chiral Chromium Salen Complexes Covalently Bound to Solid Silicates for the Enantioselective Epoxide Ring Opening. Appl. Catal. Gen..

[59] Park J.H., Bolan N. (2013). Lead Immobilization and Bioavailability in Microbial and Root Interface. J. Hazard. Mater..

[60] Bridges C.C., Zalups R.K. (2005). Molecular and Ionic Mimicry and the Transport of Toxic Metals. Toxicol. Appl. Pharmacol..

[61] Pagano G., Guida M., Tommasi F., Oral R. (2015). Health Effects and Toxicity Mechanisms of Rare Earth Elements—Knowledge Gaps and Research Prospects. Ecotoxicol. Environ. Saf..

[62] Wiche O., Zertani V., Hentschel W., Achtziger R., Midula P. (2017). Germanium and Rare Earth Elements in Topsoil and Soil-Grown Plants on Different Land Use Types in the Mining Area of Freiberg (Germany). J. Geochem. Explor..

[63] Du Laing G., Rinklebe J., Vandecasteele B., Meers E., Tack F.M.G. (2009). Trace Metal Behaviour in Estuarine and Riverine Floodplain Soils and Sediments: A Review. Sci. Total Environ..

[64] Alfassi Z.B. (1992). , Wai, C.M., Eds. Preconcentration Techniques for Trace Elements.

[65] China National Analysis Center for Iron and Steel Certificate of Certified Reference Materials LGC Standards 2021. https://bit.ly/3A7OQiC.

[66] Cao X., Chen Y., Wang X., Deng X. (2001). Effects of Redox Potential and PH Value on the Release of Rare Earth Elements from Soil. Chemosphere.

[67] Olaniran A., Balgobind A., Pillay B. (2013). Bioavailability of Heavy Metals in Soil: Impact on Microbial Biodegradation of Organic Compounds and Possible Improvement Strategies. Int. J. Mol. Sci..

[68] Tóth G., Hermann T., Da Silva M.R., Montanarella L. (2016). Heavy Metals in Agricultural Soils of the European Union with Implications for Food Safety. Environ. Int..

[69] Krachler M., Mohl C., Emons H., Shotyk W. (2002). Influence of Digestion Procedures on the Determination of Rare Earth Elements in Peat and Plant Samples by USN-ICP-MS. J. Anal. At. Spectrom..

[70] Caporaso J.G., Lauber C.L., Walters W.A., Berg-Lyons D., Lozupone C.A., Turnbaugh P.J., Fierer N., Knight R. (2011). Global Patterns of 16S RRNA Diversity at a Depth of Millions of Sequences per Sample. Proc. Natl. Acad. Sci. USA.

[71] Weißbecker C., Schnabel B., Heintz-Buschart A. (2020). Dadasnake, a Snakemake Implementation of DADA2 to Process Amplicon Sequencing Data for Microbial Ecology. GigaScience.

[72] Callahan B.J., McMurdie P.J., Rosen M.J., Han A.W., Johnson A.J.A., Holmes S.P. (2016). DADA2: High-Resolution Sample Inference from Illumina Amplicon Data. Nat. Methods.

[73] Koster J., Rahmann S. (2012). Snakemake--a Scalable Bioinformatics Workflow Engine. Bioinformatics.

[74] Schloss P.D., Westcott S.L., Ryabin T., Hall J.R., Hartmann M., Hollister E.B., Lesniewski R.A., Oakley B.B., Parks D.H., Robinson C.J. (2009). Introducing Mothur: Open-Source, Platform-Independent, Community-Supported Software for Describing and Comparing Microbial Communities. Appl. Environ. Microbiol..

[75] Quast C., Pruesse E., Yilmaz P., Gerken J., Schweer T., Yarza P., Peplies J., Glöckner F.O. (2013). The SILVA Ribosomal RNA Gene Database Project: Improved Data Processing and Web-Based Tools. Nucleic Acids Res..

